# Unusual Presentation of a Hepatic Neuroendocrine Tumor With Elevated CEA and CA 19–9: A Case Report

**DOI:** 10.7759/cureus.52858

**Published:** 2024-01-24

**Authors:** Eli Zolotov, Anat Sigal, Sara Hazaveh, Vanisha Patel, Hongfa Zhu

**Affiliations:** 1 Internal Medicine, Hackensack University Medical Center, Hackensack, USA; 2 Pediatrics, Hackensack University Medical Center, Hackensack, USA; 3 Department of Pathology, Hackensack Pathology Associates, Hackensack, USA

**Keywords:** cirrhosis mimicry, reactive fibrosis, gastrointestinal tract neoplasm, cancer antigen 19-9, ca 19-9, carcinoembryonic antigen (cea), serum tumor markers, primary liver neuroendocrine tumor, neuroendocrine tumors (nets)

## Abstract

Neuroendocrine tumors (NETs) of the gastrointestinal tract (GIT) are rare malignancies, which may have unique presentations. The diagnostic process predominantly relies on immunohistochemical analysis. While tumor markers are extensively utilized in diagnosing and monitoring GI malignancies, their specific role in NETs has not been fully explored.

This case describes an 83-year-old male presenting with jaundice and general weakness. Diagnostic imaging through MRI and CT angiography (CTA) revealed a nodular texture on the liver's surface suggesting cirrhosis. The presence of elevated tumor markers, specifically carcinoembryonic antigen (CEA) and cancer antigen 19-9 (CA 19-9), raised suspicions of malignancy. A subsequent liver biopsy confirmed the diagnosis of small-cell high-grade neuroendocrine carcinoma accompanied by reactive fibrosis.

As per our knowledge, this case is the first recorded instance of a liver neuroendocrine tumor (NET) exhibiting elevated levels of both CEA and CA 19-9, with no abnormalities detected in the gallbladder, biliary tree, and bowel in the MRI with magnetic resonance cholangiopancreatography (MRCP) and CTA. This is an atypical presentation of a liver NET, mimicking cirrhotic liver morphology, and underscores the potential diagnostic relevance of tumor markers CEA and CA 19-9 in such cases.

## Introduction

Neuroendocrine tumors (NETs) comprise a spectrum of neoplasms displaying distinctive neuroendocrine characteristics, distinguishable through immunohistochemical staining [1]. These tumors are divided into two primary groups: well-differentiated variants, commonly referred to as carcinoid tumors, and poorly differentiated carcinomas. NET cells are capable of secreting various substances, both hormonal and non-hormonal. Among the non-hormonal substances are chromogranin A (CgA), neuron-specific enolase, pancreatic polypeptide, and pancreastatin [2]. CgA is of particular significance as it exhibits the highest sensitivity and specificity, rendering it the most frequently utilized marker for NETs [3]. Recently, the NET-related transcript-based evaluations test (NETest) has emerged as a promising new biomarker, offering advantages over CgA in its specificity and sensitivity. However, incorporating this biomarker into clinical practice presents ongoing challenges [4].

Carcinoembryonic antigen (CEA) and cancer antigen 19-9 (CA 19-9) are established markers for various gastrointestinal tract (GIT) malignancies. CEA is a recognized marker associated with colorectal cancers, while CA 19-9 is well-established as a hallmark of pancreatic malignancies [5,6]. Nonetheless, the relationship between hepatic NETs and these markers remains unexplored. 

This case presents an 83-year-old male with suspected cirrhosis based on imaging, who was found to have small-cell high-grade neuroendocrine carcinoma with reactive fibrosis of the liver with both CEA and CA 19-9 elevated.

## Case presentation

An 83-year-old male with a past medical history of prostate cancer, which was managed with androgen deprivation therapy, presented to the hospital with progressive weakness, loss of appetite, and shortness of breath upon physical exertion. Upon evaluation, his vitals revealed a heart rate of 83 beats/minute, blood pressure measured at 161/72 mmHg, oxygen saturation standing at 99% while breathing on room air, and a body temperature of 36.7°C (98°F). A clinical examination revealed a distended abdomen, jaundice, and an icteric sclera.

Initial laboratory studies on admission have been summarized in Table 1. Supplementary assessments, encompassing a hepatitis panel, anti-LKM antibody, anti-liver cytosol, antinuclear antibody (ANA), anti-SM, alpha-1 antitrypsin level, and an iron panel, were all negative. Subsequent examinations, including lipase level, alpha-fetoprotein (AFP) level, and prostate-specific antigen (PSA) level, were all within the normal ranges. 

**Table 1:** Laboratory data on admission

Variable	Reference range	Admission
Hematology		
Hemoglobin (g/dL)	13.0 - 17.0	11.5
Hematocrit (%)	36 - 46	33.6
White-cell count (per μL)	4,000 - 11,000	9,400
Neutrophils (%)	40 - 75	77
Lymphocytes (%)	13 - 43	16
Monocytes (%)	0 - 13	6
Eosinophils (%)	0 - 5	0
Platelet count (per μL)	135,000 - 430,000	251,000
Chemistry		
Sodium (mmol/L)	136 - 145	136
Potassium (mmol/L)	3.5 - 5.1	5
Carbon Dioxide (mmol/L)	22 - 29	14
Creatinine (mg/dL)	0.3 - 1.5	1.05
Aspartate aminotransferase (U/L)	5 - 34	168
Alanine aminotransferase (U/L)	0 - 55	101
Total bilirubin (mg/dL)	0.2 - 1.2	8
Direct bilirubin (mg/dL)	0 - 0.5	6
Alkaline phosphatase (IU/L)	43 - 122	355
Glucose (mg/dL)	82 - 115	135
Lactate (mmol/L)	0.5 - 2.0	9.3
Ferritin (ng/mL)	21 - 275	585.8
Urine		
Color	Yellow	Cloudy
pH	5.0 - 9.0	5.5
Specific gravity	1.001 - 1.035	1.020
Nitrate	Negative	Positive
Leukocyte esterase	Negative	Moderate
RBC count (per high-power field)	0 - 2	11 - 20
WBCs (per high-power field)	<10	>50
Bacteria	Negative	Moderate

Due to the patient's persistent complaint of dyspnea during exertion, a CT angiography (CTA) was performed, which notably ruled out the presence of pulmonary emboli though revealed a 3mm nodule in the right upper lobe of the patient's lungs. A right upper quadrant ultrasound revealed a liver with heterogeneous echotexture with nodular contour, which was suggestive of cirrhotic morphology.

**Figure 1:**
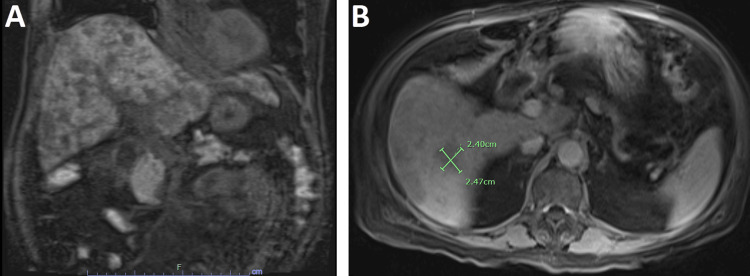
MRI abdomen with and without contrast with MRCP A: Coronal section shows nodular liver with cirrhotic morphology and normal gallbladder, biliary tree, and pancreas. B: The transverse section shows multiple hypoenhancing lesions such as the 2.4 x 2.47 cm on the image. MRCP: magnetic resonance cholangiopancreatography

A subsequent urine culture tested positive for ESBL-producing organisms, prompting the initiation of meropenem therapy, which continued for five days. On the fourth day of his hospital stay, tumor markers CEA and CA 19-9 returned with elevated levels, registering at 578 and 2117, respectively. Subsequently, an MRI of the abdomen with magnetic resonance cholangiopancreatography (MRCP) revealed a nodular liver surface contour with a normal gallbladder, biliary tree, and bowel (Figure 1).

**Figure 2:**
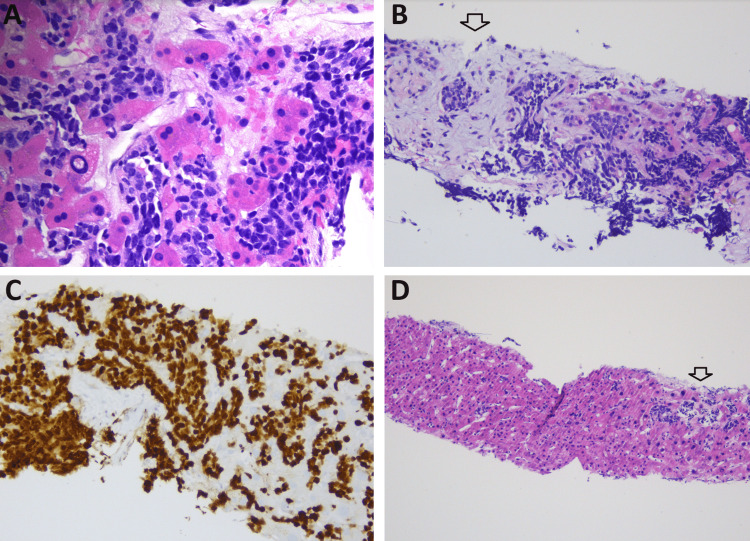
Histopathological analysis of liver tissue A: High-magnification view under H&E stain accentuates the morphology of small cell neuroendocrine carcinoma. B: H&E staining depicts infiltrative small cell neuroendocrine carcinoma with reactive myxoid stromal changes and fibrosis (indicated by the arrow). C: Ki-67 immunohistochemical staining reveals elevated index in neoplastic cells, with more than 80% of them expressing this marker. D: The H&E-stained section displays the adjacent non-tumor liver parenchyma devoid of any significant fibrotic changes, with the exception of a focal region demonstrating sinusoidal invasion by small cell neuroendocrine carcinoma (highlighted by the arrow).

Due to concerns about malignancy, a decision was made on the seventh day of hospitalization to proceed with a biopsy. The results of the biopsy unveiled a neuroendocrine carcinoma with characteristics of small-cell high-grade poorly differentiated morphology. Immunohistochemical analysis exhibited positively stained chromogranin in neuroendocrine cells, confirming their presence. The tumor displayed an aggressive nature, featuring atypical cells and a notably high proliferation rate, as indicated by the Ki67 stain showing over 80% activity. The liver parenchyma demonstrated reactive fibrosis, which can be attributed to the tissue's reparative response in proximity to the neoplastic growth (Figure 2).

Post-biopsy, the patient declined further medical intervention and was transitioned to hospice care. On day 12 post-admission, he was discharged to hospice, and on day 13, he expired.

## Discussion

We present a case that poses a unique diagnostic challenge involving a patient initially suspected of having newly diagnosed cirrhosis but ultimately found to have a neuroendocrine tumor (NET) of the liver with reactive fibrosis. Although cirrhosis has been documented in cases of liver malignancies, hepatic small-cell NETs have been associated with intra-hepatic fibrotic proliferation without evidence of cirrhosis [7,8]. This case highlights the importance of considering NETs in the differential diagnosis of patients who present with suspected cirrhotic morphology (pseudocirrhosis) on imaging [9]. Conditions resembling cirrhosis may, in fact, be due to reactive fibrosis caused by a NET.

Based on the findings from imaging and biopsy, this NET of the liver appears to be primary with evidence of local progression. It is important to interpret the positivity of thyroid transcription factor-1 (TTF-1) on the biopsy cautiously, as this marker does not definitively establish the lungs as the primary source [10]. This is especially true in the context of a benign and low-risk 3mm nodule detected on chest imaging. Furthermore, we must acknowledge that without an esophagogastroduodenoscopy (EGD) and colonoscopy, we cannot entirely rule out the presence of microfoci of malignancy within the GIT. However, it is worth noting that macroscopically, both the CT and MRI scans indicated normal bowel morphology.

In light of the patient's history of prostate cancer, the normal PSA levels suggest that prostate malignancy is less likely to be the source in this case. Nonetheless, it is important to consider that patients with progressive androgen-independent prostate cancer, unlike our current patient, may exhibit metastatic disease even when their PSA levels are low [11].

The primary novelty presented in this case revolves around the identification of elevated levels of CEA and CA 19-9 in patients with normal imaging results for the GIT and pancreas. Typically, elevated CEA is associated with pathologies in the bowel, breast, and lungs, whereas increased CA 19-9 levels are commonly observed in pancreatic cancer [12,13]. What makes this case particularly intriguing is its distinction as the first documented instance of a small cell NET of the liver co-occurring with elevated CEA and CA 19-9. After conducting an extensive literature review, these markers were generally negative in cases of primary NET of the liver [14,15]. While some articles suggest that elevated CEA and CA 19-9 may not hold significant diagnostic value in patients with neuroendocrine liver tumors [16,17], our case underscores the necessity for further investigation into the relationship between these tumor markers and neuroendocrine malignancies. Interestingly, the biopsy results from two patients with primary NETs of the liver tested positive for CEA and CA 19-9. This suggests that NETs of the liver may be associated with these tumor markers [17]. 

## Conclusions

This case presents the potential difficulty in diagnosing NETs of the liver, especially when they mimic cirrhotic morphology on imaging studies. It is important to consider NETs as a possible diagnosis in patients who appear to have cirrhosis based on their imaging results. Additionally, this is the first reported case of liver NET with concurrent elevation of both CEA and CA 19-9. Further research is essential to assess the efficacy of these tumor markers in patients diagnosed with NETs.
